# Locus Coeruleus Modulates Neuroinflammation in Parkinsonism and Dementia

**DOI:** 10.3390/ijms21228630

**Published:** 2020-11-16

**Authors:** Filippo Sean Giorgi, Francesca Biagioni, Alessandro Galgani, Nicola Pavese, Gloria Lazzeri, Francesco Fornai

**Affiliations:** 1 Department of Translational Research and New Technologies in Medicine and Surgery, University of Pisa, Via Roma 55, 56126 Pisa, Italy; giorgifs@gmail.com (F.S.G.); gloria.lazzeri@unipi.it (G.L.); 2 Neurology Unit, Pisa University Hospital, 56126 Pisa, Italy; galganialessandro@gmail.com; 3 I.R.C.C.S. Neuromed, Via Atinense 18, 86077 Pozzilli (IS), Italy; francesca.biagioni@neuromed.it; 4 Clinical Ageing Research Unit, Newcastle University, Newcastle upon Tyne NE4 5PL, UK; Nicola.Pavese@newcastle.ac.uk; 5 Institute of Clinical Medicine, PET Centre, Aarhus University, 8200 Aarhus, Denmark

**Keywords:** Locus Coeruleus, neuroinflammation, noradrenaline, Alzheimer’s disease, Parkinson’s disease, microglia, cytokines

## Abstract

Locus Coeruleus (LC) is the main noradrenergic nucleus of the central nervous system, and its neurons widely innervate the whole brain. LC is severely degenerated both in Alzheimer’s disease (AD) and in Parkinson’s disease (PD), years before the onset of clinical symptoms, through mechanisms that differ among the two disorders. Several experimental studies have shown that noradrenaline modulates neuroinflammation, mainly by acting on microglia/astrocytes function. In the present review, after a brief introduction on the anatomy and physiology of LC, we provide an overview of experimental data supporting a pathogenetic role of LC degeneration in AD and PD. Then, we describe in detail experimental data, obtained in vitro and in vivo in animal models, which support a potential role of neuroinflammation in such a link, and the specific molecules (i.e., released cytokines, glial receptors, including pattern recognition receptors and others) whose expression is altered by LC degeneration and might play a key role in AD/PD pathogenesis. New imaging and biochemical tools have recently been developed in humans to estimate in vivo the integrity of LC, the degree of neuroinflammation, and pathology AD/PD biomarkers; it is auspicable that these will allow in the near future to test the existence of a link between LC-neuroinflammation and neurodegeneration directly in patients.

## 1. Introduction

The Locus Coeruleus (LC) is the main noradrenergic nucleus in the brain. It is involved in several neuropsychological functions and in the regulation of the sleep/wake cycle. Apart from these functional effects, noradrenaline (NA) released by LC terminals exerts a variety of effects. Among them, several studies in the last decades have shown a significant modulating role on different aspects involved in neuroinflammation, with a net anti-inflammatory effect of LC-NA. Neuroinflammation is considered to play a critical role in the pathogenesis of different neurological disorders, including Alzheimer’s disease (AD) and Parkinson’s disease (PD). In these two neurodegenerative diseases (NDDs), significant degeneration of LC, which starts years before the clinical onset of the diseases, has been well documented. Such LC degeneration might concur to the pathogenesis of these NDDs through, among others, a potentiation of neuroinflammation.

In this review, we will describe and discuss the role of LC in neuroinflammation, indeed with a special emphasis on its role in NDDs. In the first paragraphs, we will provide a brief overview of neuroinflammation and describe the neuroanatomical features of LC. In the following ones, we will focus on the available experimental data supporting a role of LC in neuroinflammation and, eventually, on the available evidence on the specific involvement of LC degeneration in neuroinflammatory phenomena occurring in AD and PD.

## 2. Neuroinflammation

The term “neuroinflammation” defines the inflammatory steps taking place selectively within the central nervous system (CNS). Neuroinflammation is based on the interaction between different cell types, namely, the microglia, astrocytes, neurons, endothelium, and pericytes. All of them concur to the defense of the CNS from external *noxae*. Moreover, blood-derived circulating cells concur to neuroinflammation after getting into the CNS via the blood–brain barrier (BBB), which can be impaired primarily by the same noxae determining the neuroinflammation, or secondarily by mediators secreted by glial cells.

Despite representing by definition a mechanism of defense against external agents, neuroinflammation can concur to damaging the CNS through several mechanisms (see also [1]). For instance, there is a large amount of evidence for a significant role of neuroinflammation in the pathogenesis of degenerative disorders of the CNS, such as AD and PD, as will be mentioned in more in detail in paragraph 5.

Stepping back to neuroinflammation components, microglia play a key role in its onset and maintenance. Microglia, similarly to macrophages, are of mesodermal origin, as opposed to astrocytes, which are of neuroectodermal origin. Upon activation by different types of molecules, microglia can get a pro-inflammatory (M1) or an anti-inflammatory phenotype (M2); these can occur at different stages and time points after a same pathological event [2], and one of those can be prevalent upon the other one.

Microglia are activated by different types of ligands, mainly the pattern recognition receptors (PRRs) (which bind fragments of molecules related to pathogens), including Toll-like receptors (TLRs), as well as by other receptors such as CD-14,-33, -34, RAGE, or TREM-2 [3]. The activation of these receptors induces microglial modifications, and the secretion of different molecules, including growth factors, cytokines, and chemokines. The latter can be either anti-inflammatory (e.g., TGF-β) or pro-inflammatory ones (e.g., TNF-α, IL-6, MP-1, IL-1β), in parallel with the M2 or M1 phenotype, respectively [4].

Microglia also represent antigen-presenting cells (APCs), as they can express MHCII protein in specific circumstances, similarly to astrocytes. Indeed, it is worth noting that, even though in a very low number, T-lymphocytes can be found in brain parenchyma, and can participate in local immune response [5].

Astrocytes participate in neuroinflammatory phenomena in several ways. In particular, when stimulated by cytokines, they change their phenotype and secrete different substances including nitric oxide, cytokines, metalloproteinases, and growth factors [6]. Similar to microglia, they can also be directly activated by PRR.

One of the main characters of innate immunity is represented by the BBB integrity. It is worth noting that astrocytes also play a key role in this respect; not only do astrocyte end-feet surround the intraparenchymal vessel, forming the so-called *glia limitans*, but they also secrete substances inducing the expression of tight junctions (TJs) in endothelial cells [7]. An impairment of the BBB can allow the access of large molecules into the brain parenchyma, which can per se cause damage and contribute to triggering neuroinflammation [8].

## 3. The Locus Coeruleus

The LC is the main noradrenergic nucleus of the brain. According to the classification of catecholaminergic nuclei by Dahlström and Fuxe, LC corresponds to the A6 nucleus, which is placed in the dorsomedial part of the lateral nuclei column of the reticular formation of the pons [9]. The LC is also strictly connected with the so-called nucleus sub-coeruleus, which is placed ventrally and caudally to the main LC aggregate [10]; thus, these nuclei are often considered as part of the same structure and, in this review, we will refer to LC including both of them. LC is a tube-shaped neuronal aggregate that is placed right below the floor of the fourth ventricle at the level of the pons and extends from the posterior commissure rostrally, up to the caudal border of the pons. It is formed by a number of neurons ranging from approximately 25,000 to 50,000 in humans. The main type of neurons of LC is NA neurons, which can be further divided into two sub-types based on their shape and size. In particular, the prevalent ones are medium-size (35–45 um body diameter) neurons, each possessing several dendrites and a large axon, while a smaller type of NA neurons possesses spindle-like soma (approximately 15–20 um diameter) from whose extremities two tufts of dendrites emerge [10]. The smaller neurons are more represented in the sub-coeruleus component of LC, while the larger neurons are almost exclusively placed in the main component of LC [10]. Each of the axons originating from the medium-size LC neurons can extend for up to several cm, and it can send collaterals reaching different parts of the brain [11]. Furthermore, these axons are covered by varicosities, which represent structures from which NA can be released and affect neighboring targets through a “volume transmission”, i.e., a paracrine-type of neurotransmission [12,13,14]. At the same time, the same axons also possess a specific synaptic boutons contributing to classical synapses. Thus, a single LC neuron can simultaneously affect different parts of the brain, and this is indeed one of the most important and specific features of LC neurons.

Finally, LC NA neurons can also express co-transmitters, such as galanin, which can exert modulatory effects in post-synaptic target neurons [15]. Another typical feature of LC NA neurons is the intracellular accumulation within their cell bodies of neuromelanin (NM), a by-product of NA that can bind metal ions, and thus is considered to play a protective role towards neurons themselves, at least at the early stages of its accumulation [16]. Neuromelanin is indeed the pigment that confers the “coeruleus” (i.e., cerulean, in latin) color to the LC; it accumulates within the LC during the whole life-span, up to reaching a plateau around 60 years of age, when almost all LC NA neurons contain NM [17]; it is worth mentioning that its paramagnetic features have recently allowed the identification of LC in vivo in humans through specific magnetic resonance imaging sequences (see the review by [18]).

The LC receives afferents from a variety of structures. Interestingly, a hierarchical distribution of afferent fibers on LC neurons has been proposed, as projections from selected structures, including the *prepositus hypoglossi* and paragigantocellular nuclei, end directly in the cell body of LC medium-size neurons [19], while most afferents, originating from several, mainly sub-cortical, structures of the brain end in their distal dendrites [19,20].

LC neurons send their efferents to many cortical and subcortical structures. In particular, all cortical regions receive fibers from the LC, and in the case of the limbic cortex, such an innervation is particularly dense [21].

LC plays a key role in several physiological functions, such as attention, memory encoding, orientation to novelty, and the sleep/wake cycle [22]; furthermore, it strongly modulates neuronal plastic mechanisms in physiological [23] and pathological conditions [24,25].

Apart from such functional effects, LC terminals also play an important role in regulating the integrity of the neurovascular unit [26], and significantly modulate neuroinflammation, as will be reviewed in detail in the second part of this review.

## 4. Locus Coeruleus Degeneration in Alzheimer’s and Parkinson’s Disease

It is now well known that LC is significantly degenerated in Parkinson’s disease and Alzheimer’s disease. The earliest studies showing a marked neuronal loss in the LC of autopsies of patients with PD date back to the 1970s. Oleh Hornykiewicz himself, who is the discoverer of the occurrence of dopaminergic (DA) cell loss in the Substantia Nigra pars compacta (SNpc) in PD, described, in parallel with such SN alterations, an even more pronounced NE neuron loss in the LC in those same patients [27]. In PD patients, the number of remaining LC neurons never overlapped with the number measured in any one of the neurologically intact subjects, thus Hornykiewicz himself postulated that, in PD, LC degeneration was even as pathognomonic as the nigrostriatal dopaminergic degeneration. Such an observation was further extended by the same group, and others, in the following years, as well as by profiting from more sophisticated/quantitative histological approaches [28,29,30]. The neuronal alteration typical of PD and of other synucleinopathies is the occurrence, within the SNpc and in other brain regions, of the so-called Lewy bodies (LBs), which are neuronal inclusions formed by α-synuclein (α-syn) deposits, leading to frank cell death. In 2003, a seminal autoptic study by Braak et al. showed that LBs accumulate within the LC years before being observed at the level of SNpc [31] and that LC neuronal loss also precedes the degenerative phenomena of the SNpc by years.

In light of the early observations quoted above, several authors tried to reproduce the LC lesion in animal models of PD (such as those in which SNpc dopaminergic (DA) loss is induced by 1-methyl-4-phenyl-1,2,3,6-tetrahydropyridine -MPTP-, or by substituted amphetamine administration, in rodents or primates) in order to evaluate whether LC degeneration might concur to PD pathogenesis, rather than being just an epiphenomenon. In the early 1990s, Colpaert’s group showed that LC lesion by 6-hydroxy-dopamine in primates and by the systemic administration of N-(2-chloroethyl)-N-ethyl-2-bromobenzylamine (DSP-4) in *mice* could strongly potentiate nigrostriatal DA damage induced by MPTP [32,33]. Fornai et al. significantly extended these findings by showing the following: (a) that LC lesion makes otherwise sub-toxic doses of methamphetamine toxic for DA SNpc neurons [34] and significantly potentiates nigrostriatal loss induced by systemic methamphetamine administration in *mice* and *rats* [35]; (b) that the potentiating effects of LC lesion on DA damage in these rodent models of PD was not due to a change of MPTP/MPP+ or methamphetamine pharmacokinetics, or to an impairment of DA loss recovery, but rather to a direct potentiation of the neurotoxic effects/mechanisms of MPTP/methamphetamine themselves [34,36]. The fact that such an effect of LC pre-lesion was obtained in different animal species [34,37] and using different DA neurotoxins has been interpreted as a proof that the role of LC degeneration on the pathophysiology of nigrostriatal DA loss occurring in PD could be a sound phenomenon, which could also be extended to the human disease, according to the temporal sequence of events in which LC degeneration precedes DA loss [38].

Concerning AD, several reports obtained in small casistics dating back to the early 1980s already showed a significant neuronal loss in the LC of patients at advanced disease stage. In particular, LC degeneration was analyzed by Tomlinson et al. [39], Mann et al. [40,41], and Bondareff et al. [42], among others, and all of them showed a significant LC neuronal loss, which was proposed to more significantly affect the rostral extent of the nucleus [28]. The abovementioned studies were performed, however, in small casistics of subjects, all of which were affected by severe dementia, and in which diagnostic criteria were significantly different from the current ones. Only very recently has such evidence eventually been confirmed and extended in a seminal paper [43], in which the authors profited from large case series, from more detailed diagnostic criteria, and from stereological analysis of brain specimens, the latter providing a precise estimate of the absolute number of neurons in LC. In particular, Kelly et al., in 2017 analyzed post-mortem the brains of subjects who had been followed-up in detail in terms of neuropsychological and clinical features during their life [43]. They showed the following: (a) in patients with mild cognitive impairment (MCI) due to AD (i.e., who bear the pathological features of AD, but have only isolated episodic memory impairment and lack any need of support for daily life activities, and will eventually develop dementia due to AD), there is already a significant neuronal loss in the LC; (b) such a neuronal loss is much higher in AD dementia patients; and (c) the number of NA LC neurons is directly related to the performances in several cognitive tasks and to the global cognitive score [43].

As mentioned, in PD, the degeneration of LC is associated to the accumulation of inclusions of α-synuclein [31]. Conversely, recent studies have clearly shown that, in the LC of AD patients, there is a massive accumulation of hyperphosphorylated Tau (p-Tau) [44]. In particular, in 2011, Braak et al. [44] analyzed hundreds of brains of subjects showing different degrees of AD pathology and showed that the progressive accumulation of p-Tau in LC precedes by years the occurrence of neurofibrillary tangles (NFT) deposits in the entorhinal cortex (which was classically considered, up to that study, as the first cortical site involved by Tau pathology in AD). These authors also showed that, in AD, such early involvement of LC is related mainly to the accumulation of p-Tau (also defined by the authors as “pre-tangles”) within LC neuron perykaria, and that this also progressively extends to LC terminals, and eventually leads to frank LC neuronal loss due to NFT formation.

The effects of an LC lesion in transgenic models of AD have been investigated by several authors. The lesion of LC by DSP-4 has been shown to exacerbate dramatically the deposition of amyloid and cognitive impairment in these AD models [45,46]. Most of them also addressed the modulatory role of LC in the neuroinflammatory phenomena involved in amyloid plaques deposition; they will be described in detail below.

## 5. The Role of Neuroinflammation in Neurodegenerative Disorders

As said, neuroinflammation is a process that takes place after neuronal damage, with two main effects: removing the cause of the injury and repairing the damaged tissue. In NDDs, such a defensive mechanism can turn into a vicious cycle that causes further detrimental consequences; indeed, in NDDs, the *noxa* responsible for pathological changes cannot be removed, producing a permanent and aberrant activation of neuroinflammation, without the possibility of restoring physiological conditions.

In the last century, the occurrence of post-encephalitic parkinsonism suggested a possible link between NDDs and neuroinflammatory phenomena [47,48]. Such a hypothesis was then strengthened by experimental and post-mortem histological studies, in both AD [49,50,51] and PD [52,53,54]. In recent years, neuroinflammation has been receiving growing attention, both as a possible pathogenic element and as a therapeutic target; in line with this, neuroimaging and laboratory biomarkers of neuroinflammation are under investigation [55].

### 5.1. In Vivo and Post-Mortem Evidence for the Occurrence of Neuroinflammation in PD and AD in Humans

Positron emission tomography (PET) with 18 KDa translocation protein (TSPO) tracers is a promising tool for assessing neuroinflammation in patients, and it is widely used in pre-clinical studies [56]. TSPO is a translocator protein of the outer mitochondrial membrane, whose concentration is increased in activated microglia, thus its occurrence in brain structures has been considered as an indirect sign of inflammation mainly in relation to microglia activation [57]. Profiting from this technique, a variety of studies showed increased brain uptake of such radioligand in AD patients when compared with healthy age-matched controls [58,59,60]. Moreover, the level of the radiotracer uptake was observed to correlate with disease progression [58,59] and cerebral amyloid load [60]. Similarly, in PD patients, an increased radiotracer binding was found in basal ganglia, substantia nigra, and fronto-temporal cortex [61,62,63].

Apart from neuroimaging, several blood and cerebrospinal fluid (CSF) biomarkers are currently under evaluation, and it is worth noting that many of them are cytokines [55]. Even if some of them appear to be promising candidates for future diagnostic assays, a coherent and complete interpretation of these data is a hard task. A huge amount of studies have been performed in order to assess the blood or CSF level of interleukins and chemokines in AD, with results of non-univocal interpretation, because it seems that the cytokines’ levels widely vary, depending on the stages and specific features of the disease [64]. However, because an accurate description of cytokines as diagnostic biomarkers is beyond the aim of this review; for further details, we refer to other papers, such as [55,64].

The abovementioned data clearly show the in vivo occurrence of neuroinflammatory phenomena in patients suffering from NDDs and such a link is further corroborated by histological *post-mortem* data. In AD brain, activated microglial cells surround amyloid plaques (APs) becoming engulfed by undigested amyloid peptides [65]; they have an ameboid shape, without the characteristic ramifications of resting microglia, and are surrounded by reactive astrocytes [66,67]. Similarly, activated microglia and reactive astrogliosis associated with LB and α-syn accumulation in SNpc and in basal ganglia have been observed in PD [68].

### 5.2. Potential Neuroinflammatory Mechanisms in NDD

Many hypotheses have been proposed to explain the occurrence of neuroinflammation in NDDs, and most of them include the accumulation of aberrant proteins as one of the main potential culprits [69,70].

#### 5.2.1. Potential Mechanisms in AD

In AD, amyloid deposition is a key feature of the pathogenetic process. Soluble amyloid oligomers (AOs) accumulate, both within and outside neurons; later on, AOs aggregate into insoluble amyloid fibrils (AFs), which precipitate in the extracellular matrix, forming APs. It is not known yet whether the cause of such an alteration is more related to an uncontrolled production of AOs or to their insufficient clearance [71,72]. In any case, microglia may have a crucial role in amyloid deposition, based on two main pieces of evidence: (a) microglia is involved in amyloid clearance; (b) amyloid accumulation leads to microglia activation and inflammatory changes [73]. In physiological conditions, microglia contributes to proper functioning of synapses, mainly by removing waste by-products and releasing neurotrophic factors, which promote synaptic plasticity [2]. In particular, microglia can uptake AOs and concur to their degradation through proteases such as insulin degrading enzyme and neprilysin [74]. In AD, these clearance pathways are hindered at several levels [73]; because AFs are resistant to microglial proteases, they cannot be degraded and start accumulating within cytoplasm of microglial cells, which soon become engulfed and dysfunctional [75]. At the same time, several studies suggest that a reduction of neprilysin mRNA expression and enzymatic activity occurs in AD [76]. Moreover, it has been observed that neuroinflammation itself may dampen microglial amyloid clearance; in line with this, it has been shown that the amyloid β (Aβ)-dependent activation of the NLRP3 inflammasome is linked to reduced microglial efficiency in amyloid scavenging [77]. Thus, a vicious circle might occur in which, while amyloid accumulation evokes neuroinflammation, the latter downregulates amyloid clearance itself. The gene *TREM2* may be considered as a further proof of this phenomenon [78]. *TREM2* encodes for an immunoglobulin-like receptor, which is responsible for microglial Aβ phagocytosis and related neuroinflammatory activation [79]. In animal models carrying the human *TREM2* mutation, an increased amyloid deposition and a more severe neuroinflammation have been observed [80]. Genome-wide studies showed that subjects carrying a *TREM2* polymorphism that impairs receptor functioning have a risk of developing AD, to an extent even similar to the one related to *APOE-E4* allele (which had been considered by far as the most relevant AD risk factor) [80].

More in general, microglia recognize AFs and APs as PAMPs (i.e., pathogen-associated molecular patterns), thus they start releasing pro-inflammatory cytokines (i.e., IL1, IL6, TNF-α) and their phenotypes change from the “resting” one (i.e., M2) to the “activated” one (i.e., M1). Those released cytokines are toxic for neurons, as they impair neuronal functioning, promote apoptosis pathways, and cause reactive oxygen species (ROS) production [81]. Moreover, cytokines can activate other microglial cells and promote mononucleate cells diapedesis from blood circulation [70]. As a consequence, microglia and phagocytes produce and release harmful enzymes, such as collagenase and metalloproteinase, which lead to extracellular matrix destruction and BBB disruption, thus further impairing amyloid clearance and exacerbating the abovementioned process [82,83].

Moreover, complement activation may contribute to this process; complement factors can bind AFs and APs, mediating their phagocytosis by microglial cells [84]. At the same time, the release of complement-derived factors, namely C3a and C5a, promotes microglial activation, cytokines, and ROS production. In AD brains, the occurrence of higher levels of C1q and C3 factors was observed and related to amyloid pathology [85].

Apart from Aβ, neuroinflammation occurring in AD has also been related to tau pathology. P-Tau and NFTs promote microglial activation and inflammatory cytokines production. Moreover, it has been observed that tau is associated with T-lymphocytes recruitment, and this might represent a possible link between adaptive immune response and AD pathology [86].

#### 5.2.2. Potential Mechanisms in PD

In PD, α-syn may play an important role in evoking neuroinflammation [69]. In animal models carrying the human gene for α-syn, the authors observed microglial activation, lymphocytes infiltrations, and increased expression of pro-inflammatory cytokines and chemokines; similar observations were obtained in animals in which α-syn was injected directly into the brain [87]. At the same time, interleukins and other neuroinflammation promoters have been shown to promote α-syn accumulation and LB formation [88].

The relationship between PD and neuroinflammation goes far beyond the occurrence of α-syn accumulation. Dopaminergic neurons have been found to be very sensitive to inflammatory damage; in animal models exposed to lipopolysaccharide (LPS), a molecule causing a strong systemic inflammatory reaction, a significant degeneration of DA brain cells was observed [89]. Such a vulnerability could be explained not only in light of the neurotoxic effects of cytokines released by microglia, but also considering the large amount of ROS produced by the latter; indeed, DA cells have been shown to be particularly sensitive to oxidative stress, as they lack strong anti-oxidant defense mechanisms [90]. Furthermore, it is worth noting that two of the genes linked to genetic PD, i.e., *Parkin* and *LRRK2*, encode for proteins involved in the regulation of microglia activity; in particular, *Parkin* mutation is associated with a more severe damage of DA neurons and increased production of M1 cytokines [91], while LRRK2 is expressed in activated microglia and takes part in the modulation of inflammation in response of pathological stimuli [92].

## 6. The Potential Role of LC Degeneration in Neuroinflammation Promotion during NDD Pathogenesis

As mentioned above, LC degeneration occurs early in the pathogenesis of both AD and PD and the impairment of the LC-NA system may significantly contribute to the pathological processes; in particular, several pieces of evidences have associated LC impairment with increased neuroinflammation and, at the same time, a variety of NA-dependent cellular mechanisms involved in such a link have been identified [93].

### 6.1. General Effects of Locus Coeruleus on Neuroinflammation

Experimental studies have shown that the central NA system may exert an inhibitory control on neuroinflammation, activating both anti-inflammatory and neuroprotective cellular pathways. LC-NA could reduce the production of pro-inflammatory interleukins, such as TNF-α [94] and MCP-1 [46], as well as promote the expression of the anti-inflammatory IL-10 [95]. At the same time, LC modulates the activation of microglia and astrocytes, stabilizing these cells in their resting state through the activation of anti-inflammatory mediators, such as peroxisome proliferator-activated receptor gamma (PPARγ) [96] or the heat shock protein 70 (HSP70) [97]; in addition, NA has an inhibitory effect on NF-Kβ, which is responsible for the activation of inflammatory response in microglial cells [98]. NA reduces the expression of the type 2 major complex of histocompatibility (MCH-II) in astrocytes, activated with IFN-gamma [99]; moreover, it may inhibit the production of the inducible enzymes NOS and COX-2, in both astrocytes and microglial cells [98].

In animal models of NDD, the experimental lesion of LC triggers neuroinflammation and is associated with an increased neuropathological burden. These studies were performed mainly by profiting from the LC-specific neurotoxin DSP-4, a compound that selectively targets LC-NA cells, sparing other monoaminergic neurons [37,100] (Table 1). Furthermore, indirect data on the effects of LC stimulation have been obtained in experimental studies on NDD in which NA/NA agonists were administered, as most of brain NA originates from the LC (Table 1).

### 6.2. Experimental Data on the Role of Locus Coeruleus in Neuroinflammation Occurring in Alzheimer’s Disease

In AD, LC-NA modulates the neuroinflammatory response to amyloid accumulation, also promoting Aβ-42 clearance via microglial phagocytosis. Such an effect has been widely studied by Heneka’s group, which assessed the effect of LC lesion in animal models of AD. In 2002, they observed that the intra-cortical injection of Aβ-42 aggregates causes a more severe neuroinflammatory reaction in the brain of LC-lesion rats compared with LC-intact animals; in particular, they found increased expression of IL-1β, IL6, and NOS genes by astroglial cells [101]. Such hyperreactivity was associated by the same authors with the reduction of NF-kβ inhibitory protein (Ikβ) and HSP-70 levels that occur after the lesion of LC [102].

However, interestingly, in another study, it was observed that, when neuroinflammation is evoked by chronical infusion of TNF-α, the lesion of LC by DSP-4 does not produce significant modifications in neuronal degeneration in rats [103].

Nonetheless, in order to evaluate the NA role in a pathophysiological model of AD, in 2006, Heneka and coll. lesioned the LC of mice transgenic for the amyloid precursor protein 23 (APP23); they observed that, in animals lacking LC-NA, the amyloid and neuroinflammatory burdens increased significantly when compared with control animals. As a further proof of concept, such alterations were not detectable in brain areas that do not receive LC-projections, like the paraventricular thalamus, thus strengthening the association between NA and neuroinflammation [45]. As said, the LC-NA loss is also associated with increased amyloid accumulation; this phenomenon is probably due to the impairment of amyloid microglial phagocytosis. In 2010, Heneka and collaborators observed that, in LC-damaged AD transgenic mice, the accumulation of Aβ was associated with aberrant activity of microglial cells; indeed, in the absence of NA, microglia reduced the efficiency of amyloid phagocytosis and its recruitment at the level of the amyloid plaque [104]. Other authors obtained similar results [105,106,107,108] (see Table 1).

Intriguingly, microglial dysregulation may not be the only consequence of LC degeneration responsible for increased amyloid accumulation and neuroinflammation; several pieces of evidence suggest that LC-NA plays an important role in maintaining the homeostasis of the BBB [26]. BBB impairment is considered to be a key feature of AD pathology and to play an important role in its pathogenesis; indeed, as a large amount of amyloid is eliminated through trans-endothelial transport from interstitial space to blood flow, BBB breakdown may cause increased amyloid accumulation [83]. Moreover, damaged BBB allows several plasmatic proteins to extravasate from leaky capillaries and to accumulate in the extravascular matrix; among these, it is worth mentioning thrombin, plasmin, and complement factor, all of which have been shown to evoke neuroinflammation [8]. LC promotes endothelial TJ expression [109], thus regulating BBB transcytosis [110] and modulating water and ionic flow [111]; in line with this, LC degeneration occurring in AD may also lead to further amyloid deposition and neuroinflammation promotion through BBB impairment [26].

The analysis of the effects of pharmacological modulation of the LC-NA system gave contrasting results. In one study, Scullion et al. (2011) indirectly stimulated NA activity by administering an α-2 antagonist, i.e., fluparoxan, to *APPxPS1* transgenic mice, and they did not observe significant differences between treated and control animals, in terms of both amyloid or neuroinflammatory burden [112]. On the contrary, the administration of reboxetine, an NA reuptake inhibitor, was shown to reduce microglial activation, astrogliosis, and amyloid accumulation [113].

Apart from the above mechanisms, more recent studies have also assessed the role of tauopathy in LC degeneration itself and related neuroinflammation in AD models. In fact, as already described in paragraph 3, according to Braak staging, the first AD-related pathological changes occur in the LC and are represented by p-Tau accumulation within LC-NA neurons [44]. Tau-related pathology is considered to be responsible for the LC impairment in AD [114], and it may spread to cortical and other sub-cortical brain structures through LC axonal fibers themselves [115,116]. In 2016, Mravec and colleagues assessed LC involvement and neuroinflammatory changes in a rat model of human tauopathy; they observed the occurrence of LC degeneration together with decreased NA levels. Such alterations were associated with increased expression of pro-inflammatory mediators like IL-6, TNF-α, and NOS and correlated with NA levels [117]. A more specific study was performed by Chalermpalanupap et al., which lesioned the LC of tau transgenic mice by DSP-4; at the level of the hippocampus of lesioned animals, they found a greater burden of tau-related pathology, together with higher levels of microglial activation, when compared with LC-intact animals [118].

Finally, it is worth mentioning the only study in which the effect of DSP-4 lesion on neuroinflammation was assessed in non-human primates; in 2019, Duffy and colleagues administered DSP-4 to normal macaques, which were then sacrificed months later. The authors observed increased amyloid burden in the hippocampus and in the frontotemporal cortex, associated with a higher level of Aβ-42 production, but they did not observe a significant activation of microglia, neither in the control nor in the lesioned animals [119].

### 6.3. Experimental Data on the Role of Locus Coeruleus in Neuroinflammation Occurring in Parkinson’s Disease

Regarding PD, the protective role of LC-NA on nigrostriatal DA cells has been known for decades [38]. As described in detail in paragraph 3, in an experimental model of PD, LC lesion was associated with increased DA cells sensitivity to damage [120] and with a higher degree of nigrostriatal degeneration [36]. Recently, such a protective effect has been linked to neuroinflammatory pathways’ modulation and anti-oxidant mechanisms. In particular, in vitro studies showed that, in mesencephalic DA cultures, the administration of NA reduces the production of reactive oxygen species and neuronal death rate [121]. Similar results were observed in animal models; in a paraquat/maneb PD mice model, LC lesion obtained by DSP-4 administration increased neuroinflammation burden and exacerbated oxidative stress, as shown by reduced levels of glutathione peroxidase and by an increased concentration of lipid peroxidation byproducts [122].

LC damage was found to also increase neuroinflammatory damage in other experimental models. In rats, DSP-4 treatment has been associated with subsequent nigral DA cells’ degeneration and a high level of microglial activation in the striatum [123]. Because systemic inflammation may play a key role in PD pathogenesis [89], some authors assessed the effects of LPS administration in models of PD susceptibility [124]. In LPS-treated mice, the occurrence of nigrostriatal, hippocampal, and motor cortex degeneration was exacerbated by DSP-4-induced LC lesion; neuronal damage was accompanied by increased microglial activation and neuroinflammatory reaction [125,126,127].

At the same time, it has been suggested that indirect LC-NA stimulation may reduce neuroinflammation and DA damage. Such pieces of evidences have been obtained in animals submitted to vagal nerve stimulation (VNS). VNS is known to exert an activating effect on LC, through the cholinergic projections the nucleus of solitary tract sends to it [128]. In line with this, in PD models, VNS reduced glial activation and α-syn accumulation, in both LC-lesioned and LC-intact rats [129,130].

## 7. Discussion and Conclusions

**Figure 1:**
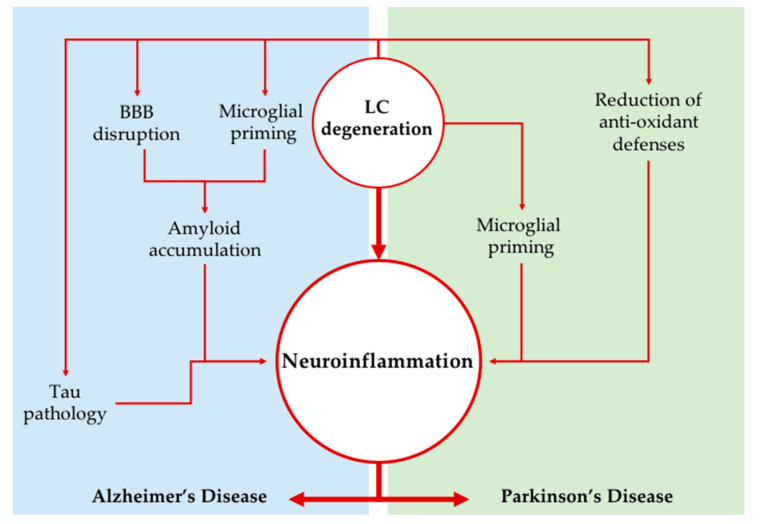
Locus Coeruleus degeneration as a driver of neuroinflammation. The impairment of the LC-NA system may promote neuroinflammation in neurodegenerative disorders through several mechanisms; the loss of NA modulation causes microglial cells to change their phenotypes into a more reactive one. Moreover, in Alzheimer’s disease, NA impairment concurs to BBB breakdown and amyloid and phospho-tau accumulation (Tau pathology), which together further exacerbate neuroinflammatory phenomena. On the other hand, in Parkinson’s disease, increased oxidative damage related to inflammation has been observed after LC lesion. BBB: blood brain barrier; LC: Locus Coeruleus; NA: noradrenaline.

The LC loss occurring in AD and PD, apart from contributing to some of the signs and symptoms occurring in the two NDDs (e.g., sleep/waking alterations, memory/executive function complaints, impairment of attention, and hypotensive states), is likely to contribute dramatically to the pathogenesis of the two disorders, as shown by several studies in experimental animals. An increase in neuroinflammation has been shown to play a potential key role in such a contribution, and this might be especially true concerning the potentiation of amyloid burden in AD. Several experimental models showed a specific strong role of LC in neuroinflammation through modulation of microglia and astroglia activity (summarized in Figure 1). There is also evidence for a significant role of complement-mediated neuronal death in AD pathogenesis, but to the best of our knowledge, the role of LC on this phenomenon has not been explored in detail yet.

The fact that the role of LC in neuroinflammation has been confirmed in different models and by a variety of approaches (again, especially concerning AD) suggests that this phenomenon is likely to also occur in humans. However, experimental models obviously bear intrinsic limitations. For instance, one could not completely exclude that the direct neurotoxic effect of DSP-4, or other toxins used to induce LC lesion, may already contribute by itself, at least in part, to the neuroinflammation increase observed after LC lesion.

Thus, it appears mandatory to also obtain direct confirmations of the role of LC in neuroinflammation in PD/AD in patients in vivo. Potentially, nowadays, there are a variety of experimental tools that can be used in humans to assess neuroinflammation, as well as tools that allow estimating in vivo LC integrity. In particular, CSF analysis is currently performed in most subjects with suspect degenerative dementia, in order to assess the profile of amyloid/p-tau/total tau in AD/MCI patients, as well as in subjects affected by other NDDs; in this same biological matrix, the concentration of different types of inflammatory/anti-inflammatory cytokines can also be directly assessed, which allows an estimation of their concentration in the brain. Again, as mentioned in paragraph 5, nowadays, it is possible to directly estimate in vivo, in humans, the burden of microglia-related neuroinflammation by TSPO-ligand PET tracers. These approaches have shown increased neuroinflammation in the brain of PD and AD patients [57], but they have not yet been put in relation with LC markers obtained in the same subjects.

In humans, LC features can be estimated non-invasively by specific MRI T1-wighted sequences and ad-hoc post-processing analysis. These MRI tools, the development of which started in the last decades, have been progressively refined, up to recent, more sophisticated approaches allowing to estimate not only the LC cell density, but also its volumetric features [131]. By the same token, PET tracers specific for noradrenergic transporters have recently been developed [132]. Finally, it has been possible to quite reliably directly assess the levels of amyloid and tau-related pathology in vivo in patients through PET or CSF analysis for almost 10 years, and these are nowadays assessed almost routinely in memory clinics to confirm AD phenotype in single patients [133,134,135]. Thus, it is auspicable that, in the future, the link between the loss of LC integrity and neuroinflammatory burden, in relation to AD (and PD) pathology, could be tested directly in patients in vivo.

Apart from the direct assessment of such a pathogenetic link, early identification of the combination of these biomarkers (i.e., LC parameters, neuroinflammation, and amyloid/tau/syn parameters) in a patient might theoretically even allow to directly intervene at the early stages of NDD in order to slow its progression. More in detail, one might try to intervene (a) on the mechanisms causing LC degeneration and/or (b) to replace pharmacologically the noradrenergic tone, which is dramatically reduced in LC target structures after LC degeneration.

Concerning the first aspect, i.e., trying to intervene as soon as possible on the degenerative processes causing NA neuronal loss, it is worth noting that the molecular mechanisms involved in the degeneration of LC in PD and AD are likely significantly different from one another. In fact, LC degeneration in PD is mainly due to the accumulation of α-syn, up to LB formation [31], while in AD, it degenerates after the progressive accumulation of p-Tau within NA cell bodies, up to frank accumulation of NFT within the axons and cell bodies [44]. Unfortunately, the precise mechanisms through which both pathological processes occur are not clear yet (a detailed description of them is beyond the aim of this review), and thus it is unlikely that in the near future there will be promising therapeutic tools aimed at halting LC degeneration.

Conversely, a promising approach might be represented by replacing the impaired NA tone early in the course of NDD. In line with this, there are several pieces of evidence obtained in experimental models in vitro and in vivo, in which the effects of adrenergic agonists on neuroinflammation have been assessed, and some of them were obtained in the context neurodegenerative phenomena. NE induces the expression of several anti-inflammatory genes (including IL-10, heat shock protein 70, and PPARγ) in both neurons and glia [95,96,97]. Furthermore, NE has been shown to decrease, through β-AR activation, the microglial activity of nuclear factor kappa-light-chain enhancer of activated B cells (NF-kB), which is involved in the transcription of pro-inflammatory molecules such as IL-8 and TNF- α [136]. The latter is indeed reduced by NE in microglia [46].

More specifically, in the context of AD pathogenesis, in rats pre-treated with DSP-4 and submitted to cortical microinfusion of Aβ1-42, it has been shown that the increased levels of neuronal iNOS and of IL1β expression in microglia were dramatically attenuated by co-injection of the β2-AR agonist isoproterenol and of NE [101]. In *APP*-transgenic mice, NE application has been shown to enhance microglial migration and Aβ clearance [46,105,106,107,108,137]. In in vitro experiments in which microglia was exposed to Aβ, NE application prevented the Aβ-related production of chemokines and cytokines and the induction of pro-inflammatory genes such as *TNF-α, iNOS, CCL-2*, and *MCP-1*, and at the same time, β2-AR stimulation increased Aβ phagocytosis and Aβ-related microglial migration [46].

Less clear evidence for a beneficial effect of AR stimulation on the neuroinflammatory phenomena occurring in PD models is available; however, recent exciting data on a potential beneficial effect on PD of β-AR agonists have been obtained based on data analysis of clinical records available for the entire Norwegian population by the Norwegian National Registry and the Norwegian Prescription Database [138]. In this study, Mittal et al. retrospectively extrapolated a potential significant protective effect of treatment with β2-AR agonists on PD. Even though they also showed that β2-AR agonists reduce the expression of synuclein, in the same paper, the authors themselves also discussed the potential role of β2-AR protective effects in light of the anti-inflammatory role [138].

Thus, there are at least some hints, concerning both NDDs, for a potential beneficial role of β2-AR agonists on the neuroinflammatory mechanisms involved in degenerative phenomena, and drugs with such mechanisms might be worth testing. Furthermore, one might also assess potential beneficial effects of an increase of NE in the brain; in line with this, it is worth mentioning the study by Gutierrez et al. [113], who showed that reboxetine (a selective blocker of NA reuptake) was able to reduce neuroinflammation and neurodegeneration in the 5xFAD mouse model of AD. With this purpose, the administration of synthetic NA precursors, such as L-threo-3,4-dihydroxyphenylserine (which is able to selectively increase NA levels in the brain), might also represent an interesting therapeutic approach to be tested in the future [98,139]. Finally, it is worth mentioning that VNS, which is a therapeutic tool already approved in humans for the treatment of specific types of epilepsy and severe depression [140,141], is known to exert an indirect strong activating effect on LC [128] and reduces glial activation and α-syn accumulation LC-lesioned animals [129,130]. It is worth noting that there have been already proposals for the use of VNS in AD and PD patients as well [142,143], and this might represent indeed a useful approach for an early NE-related modulation of neuroinflammation in these NDDs.

## Figures and Tables

**Table 1:** Experimental evidences for a role of Locus Coeruleus in neuroinflammatory changes occurring in Parkinson’s disease and Alzheimer’s disease.

Authors and Year [Ref.]	Disease	Animalspecies	LC Manipulation	Neuroinflammatory Changes
Heneka et al. 2002	AD	*Rats*	DSP-4 lesion	Increased inflammatory response after intracortical amyloid peptides injection
Heneka et al. 2003	AD	*Rats*	DSP-4 lesion	Reduced basal levels of NF-kβ inhibitory Ikβ protein and expression of HSP-70
Wenk et al. 2003	AD	*Mice*	DSP-4 lesion	TNF-α injection within BF caused cholinergic cells death and evoked neuroinflammation, with no differences between LC-lesioned and control mice
Heneka et al. 2006	AD	*APP23* Tg *Mice*	DSP-4 lesion	Increased amyloid deposition and elevation of microglial/astrocytic activation
Kalinin et al. 2007	AD	*APP V717F* Tg *Mice*	DSP-4 lesion	Increased amyloid deposition and astroglial activation
Pugh et al. 2007	AD	*APP/PS1* Tg *Mice*	DSP-4 lesion	Increased level of IL-1β and reduced level of NF-kβ inhibitory Ikβ protein
Heneka et al. 2010	AD	*APP V717F* Tg *Mice*	DSP-4 lesion	Increased amyloid deposition—independently of APP processing; increased pro-inflammatory chemokines and cytokines concentration; impaired microglial amyloid clearance
			L-threo-DOPS	Restoration of microglial activity and reduction of cytokines production
Jardanhazi-Kurutz et al. 2010	AD	*APP/PS1* Tg *Mice*	DSP-4 lesion	Increased amyloid accumulation and macrophages activation
Jardanhazi-Kurutz et al. 2011	AD	*APP/PS1* Tg *Mice*	DSP-4 lesion	Increased amyloid accumulation and elevation of IL-1β level
Scullion et al. 2011	AD	*APP/PS1* Tg *Mice*	Fluparoxan	The administration of the α-2 blocker was associated with better cognitive performances, while it did not reduce neuroinflammatory or amyloid burden
Mravec et al. 2016	AD	*SHR72* Tg *Mice*	\	Induced tauopathy causes LC degeneration, which is associated with reduction of NA level and increased concentration of IL6, TNF-α, and iNOS
Chalermpalanupap et al. 2018	AD	*P301S* Tg *Mice*	DSP-4 lesion	Increased tau pathology burden in the hippocampus, accompanied by higher microglial activation
Gutierrez et al. 2019	AD	*5xFAD Mice*	Reboxetine	Reduced neuroinflammatory and amyloid burden
Bharani et al. 2017	\	*Rats*	DSP-4 lesion	Increased astroglial activation and serum interleukin concentration after LPS exposure
Farrand et al. 2017	PD	6-OHDA treated *Rats*	DSP-4 lesion/VNS	Both in DSP-4 lesioned and control rats, VNS was associated with better motor performance, reduced neuroinflammation, and increased NA markers in LC, SN, and striatum
Bjerken et al. 2019	PD	*Rats*	DSP-4 lesion	LC degeneration caused DA cell degeneration; activated microglial observed in impaired substantia nigra
Hou et al. 2019	PD	PQ/MB treated *Mice*	DSP-4 lesion	Increased degeneration of DA cells; reduction of glutathione contents; increased microglia activation and M1 polarization
Song et al. 2019 (a)	PD	*gp91phox−/− Mice*	DSP-4 lesion	Increased microglial activation and oxidative stress in DA cells
Song et al. 2019 (b)	PD	*Mice*	DSP-4 lesion	Increased neurodegeneration, neuroinflammation, and oxidative stress induced by LPS exposure
Farrand et al. 2020	PD	6-OHDA treated *Rats*	DSP-4 lesion/VNS	High frequency VNS was associated with better motor performances, reduced glial activation, and NA impairment

The table reports experimental evidences for a role of Locus Coeruleus in neuroinflammatory changes occurring in Parkinson’s disease and Alzheimer’s disease. PQ: paraquat; MB: maneb; VNS: vagus nerve stimulation; AD: Alzheimer’s disease; PD: Parkinson’s disease; DSP-4: N-Ethyl-N-(2-chloroethyl)-2- bromobenzylamine hydrochloride; L-threo-DOPS: L-threo-dihydroxyphenylserine; LC: Locus Coeruleus; SN: substantia nigra; BF: basal forebrain; Fluparoxan: alfa-2 antagonist; 6-OHDA: 6-hydroxydopamine; Reboxetine: selective noradrenaline reuptake inhibitor; APP: amyloid precursor protein; HSP: heat shock protein; LPS: lipopolysaccharide; NF-kB: nuclear factor kappa-light-chain enhancer of activated B cells.

## Abbreviations

Aβ	Amyloid β
AD	Alzheimer’s disease
AF	Amyloid fibrils
α-syn	α-synuclein
AO	Amyloid oligomer
AP	Amyloid plaque
APC	Antigen presenting cell
APP23	Amyloid precursor protein 23
BBB	Blood-brain barrier
CSF	Cerebrospinal fluid
CNS	Central nervous system
DA	Dopamine
DSP-4	N-(2-chloroethyl)-N-ethyl-2-bromobenzylamine
HSP-70	Heat shock protein 70
LB	Lewy body
LC	Locus Coeruleus
LPS	Lipopolysaccharide
MCI	Mild cognitive impairment
MTPT	1-methyl-4-phenyl-1,2,3,6-tetrahydropyridine
NA	Noradrenaline
NDD	Neurodegenerative disorders
NFT	Neurofibrillary tangle
NM	Neuromelanin
PAMP	Pathogen-associated molecular pattern
PD	Parkinson’s disease
PET	Positron emission tomography
PPARγ	Peroxisome proliferator-activated receptor-γ
PRR	Pattern recognition receptor
p-Tau	Hyperphosphorylated tau
ROS	Reactive oxygen species
SNpc	Substantia nigra pars compacta
TJ	Tight junction
TLR	Toll-like receptor
TSPO	Translocation protein
VNS	Vagus nerve stimulation.
